# Avatar-based versus conventional vital sign display in a central monitor for monitoring multiple patients: a multicenter computer-based laboratory study

**DOI:** 10.1186/s12911-020-1032-4

**Published:** 2020-02-10

**Authors:** Olivier Garot, Julian Rössler, Juliane Pfarr, Michael T. Ganter, Donat R. Spahn, Christoph B. Nöthiger, David W. Tscholl

**Affiliations:** 1 Institute of Anesthesiology, University and University Hospital Zurich, Raemistrasse 100, 8091 Zurich, Switzerland; 2 Institute of Anesthesiology and Pain Therapy, Cantonal Hospital Winterthur, Winterthur, Switzerland

**Keywords:** Patient monitoring, Patient safety, Signal processing, Situation awareness

## Abstract

**Background:**

Maintaining adequate situation awareness is crucial for patient safety. Previous studies found that the use of avatar-based monitoring (Visual Patient Technology) improved the perception of vital signs compared to conventional monitoring showing numerical and waveform data; and was further associated with a reduction of perceived workload. In this study, we aimed to evaluate the effectiveness of Visual Patient Technology on perceptive performance and perceived workload when monitoring multiple patients at the same time, such as in central station monitors in intensive care units or operating rooms.

**Methods:**

A prospective, within-subject, computer-based laboratory study was performed in two tertiary care hospitals in Switzerland in 2018. Thirty-eight physician and nurse anesthetists volunteered for the study. The participants were shown four different central monitor scenarios in sequence, where each scenario displayed two critical and four healthy patients simultaneously for 10 or 30 s. After each scenario, participants had to recall the vital signs of the critical patients. Perceived workload was assessed with the National Aeronautics and Space Administration Task-Load-Index (NASA TLX) questionnaire.

**Results:**

In the 10-s scenarios, the median number of remembered vital signs significantly improved from 7 to 11 using avatar-based versus conventional monitoring with a mean of differences of 4 vital signs, 95% confidence interval (CI) 2 to 6, *p* < 0.001. At the same time, the median NASA TLX scores were significantly lower for avatar-based monitoring (67 vs. 77) with a mean of differences of 6 points, 95% CI 0.5 to 11, *p* = 0.034. In the 30-s scenarios, vital sign perception and workload did not differ significantly.

**Conclusions:**

In central monitor multiple patient monitoring, we found a significant improvement of vital sign perception and reduction of perceived workload using Visual Patient Technology, compared to conventional monitoring. The technology enabled improved assessment of patient status and may, thereby, help to increase situation awareness and enhance patient safety.

## Background

The World Health Organization, in its guidelines for safe surgery, considers the continuous presence of a professionally trained and vigilant anesthesia provider using standardized patient monitoring to be of central importance for safe perioperative care. The range and scope of applied monitoring may vary but should match minimal requirements and never substitute clinical observation and assessment [1]. Patient monitoring displays large amounts of information and may include various acoustic and visual signals, e.g., alarm sounds, waveforms, and numbers. Anesthesia providers must invest high mental effort to observe patients and their appendant surroundings continuously, and to integrate these sensory inputs into a mental model of the current situation and the expected immediate future. A technical definition of situation awareness provided by Endsley is: “the perception of the elements in the environment within a volume of time and space, the comprehension of their meaning and a projection of their status in the near future” [2–4]. Maintaining situation awareness is an important non-technical skill as it enables informed decision making: To make a well-founded decision, a decision maker must first correctly assess the situation at hand. In the operating room and the intensive care unit, maintaining situation awareness goes beyond just patient monitoring. It is a constantly evolving multi-directional process that takes place between care providers and their environment, which may include, e.g., the patient, other team members, external and self-induced distractions. Actions and events like these, may change the care providers’ mental model and influence their further actions [5].

Errors in situation awareness can occur on all three levels: 1. perception; 2. comprehension; 3. projection, but with a predominant proportion in perception and comprehension. A subcategory of perception errors is failure to detect or perceive data, which is available in a system, e.g., overlooking a value on a patient monitor even though it is displayed [3–6]. Although monitors are a vital source of information, observational studies have found that anesthetists look at monitors in 1- to 2-s glances and overall only during about 5% of the observed time [7, 8]. It has also been found that increasing the amount of information shown on monitors reduces the ability to detect unexpected changes even when a visual event or change is in plain view [9–11]. This phenomenon is called inattentional blindness and is a major cause of situation awareness failure [12–14].

To improve situation awareness, minimize inattentional blindness, and potentially enhance patient safety, presentation of monitoring information can be redesigned. One possibility is through transformation of the multitude of numbers and waveforms of current monitors into objects and pictures, which may be simpler to interpret. One research group developed an interface that represents stroke volume and heart rate as a rectangle with variable height and width, and another group created a display that depicts pulmonary function data anatomically [15–17]. A review article concluded that object or graphical displays may result in a faster detection of changes and more successful treatment [18]. In addition to simplifying the visual presentation of monitoring information, there have been other attempts to improve users’ situation awareness. One study, e.g., analyzed a vibro-tactile-belt display, which mapped and converted monitoring parameters and their changings into stimulation patterns [19]. Another study, which evaluated the potential of a head-worn display for multiple patient monitoring in supervising anesthetists found that the device improved situation awareness [20]. The emergence of these situation awareness-based technologies represents a growing awareness of shortcomings of conventional monitoring [21].

In this study, we evaluate the usefulness of Visual Patient technology for monitoring multiple patients at the same time, as it is practiced in the modern operating room and intensive care station central station monitors. Visual Patient is an advanced patient monitoring visualization technology, which creates an animated virtual patient avatar from patient monitoring data. The hypothesis of this study was that there would be an improvement in remembered vital signs as well as in perceived workload with Visual Patient technology, analogous to the previous studies, which evaluated the effects of the technology in single patient monitoring. How the technology would behave in the specific setting of a central monitor could not, in our opinion, be deduced from the previous studies, which is why we investigate it separately in this study.

## Methods

### Visual Patient technology

We developed the Visual Patient Technology in analogy to the synthetic vision technology used in aviation. Synthetic vision renders a virtual image of the flight situation from satellite position data, aircraft attitude (orientation in space), altitude, heading, terrain and traffic data, and other data available in an aircraft. The resulting image looks to the pilots as if they looked out of the window in clear weather. A lake looks like a lake, a mountain like a mountain, etc. Therefore, the flight situation can be interpreted more intuitively than when the pilots have to compile this “image” from lower-level data in their minds, as is the case with conventional instruments. According to user-centered, situation awareness-based design principles and standard works of logic, a model is then ideal if its design has a logical relation to the reality it tries to represent, also termed direct display of information [4, 22]. Just like synthetic vision technology, Visual Patient Technology displays the monitoring information in a way that corresponds to the expected phenomena in the real patient it represents. For example, the Visual Patient avatar becomes cyanotic (skin turns purple) if oxygen saturation is low, or it emits thermal radiation from its body if the body temperature becomes too high. The realistic depiction of reality is also the reason why the patient avatar is upside down in the anesthesia view tested in this study: because this corresponds to the common viewing position of the anesthesiologist on the patient. The three main characteristics of the avatar are the pre-processing of the data, the direct presentation of the information and the integration of the vital signs in several visualizations, e.g., care providers can judge the respiratory rate on the basis of the respiratory rate of the lung as well as the formation rate of the carbon dioxide cloud exhaled by the patient model. These characteristics translate a large number of numerical values and waveforms into an animated model of the patient’s situation, which the care provider can evaluate and store at a glance without first having to read and derive meaning from data values. The translation of the vital signs from the numerical data into the avatar model takes place in real-time as with regular monitoring. Since we started developing and studying Visual Patient Technology in 2012, we found that the technology, compared to conventional number and waveform-based monitors, improved the perception of vital sign information in anesthesia providers, reduced the perceived workload, and received positive user feedback [23, 24]. Additionally, the technology improved perceptive performance in a setting with a distraction [25]. In eye-tracking studies, we found that the technology works because users do not need to read numbers sequentially but can receive information from looking at colorful moving objects, which allows for monitoring using peripheral vision and enables parallel perception of multiple vital signs at the same time [26, 27].

### Study design

This study was an investigator-initiated, within-subject, prospective, multicenter trial comparing two different multiple patient monitoring interfaces. We conducted this multicenter study in Switzerland with anesthesia providers from the University Hospital Zurich (USZ) and the Cantonal Hospital Winterthur (KSW).

### Study participants

In this study, we included resident and staff anesthesiologists and certified anesthesia nurses who worked in the two study centers at the time the study was conducted. These anesthesia providers were invited to participate and relieved from their duties for the duration of their participation on a regular working day. The participants were included according to an inclusion plan, which served to ensure that the sample included equal numbers of participants of all professional groups and genders in both centers. Eight participants in Zurich and 8 participants in Winterthur assessed the 10-s scenario and 8 participants in Zurich and 14 participants in Winterthur evaluated the 30-s scenario. The last group included more than the minimum of 8 participants required in each group because 6 additional participants were institutionally planned to participate in the study, and accordingly available on the days we tested the 30-s scenario in Winterthur. Participation in this study was voluntary and there was no monetary compensation for the participants. All participants signed a written informed consent form agreeing to the use of their data in anonymous form for scientific purposes.

### Power analysis and sample size calculation

Before the actual study, we conducted a pilot study with five participants in order to estimate the expected effect size as the basis for sample size planning. These five participants evaluated three 10-s scenarios. In this pilot study, the mean of differences between the participants’ performance with avatar and conventional monitoring was two vital signs with a standard deviation of the differences of 0.8. Using the standard deviation of the differences obtained in the pilot study and a mean of differences of one vital sign, which we considered to be the minimum clinically significant difference, an effect size of 1.25 resulted. For an alpha-error probability of 0.05 and a power of 80%, the required sample size was 8 participants. To detect a difference of one vital sign with the specified power at the 5% significance level in each center, for both scenarios, and both viewing durations, we had to include at least 16 participants per center, or 32 participants total, who each evaluated two scenarios. The computer program used for the sample size calculations was G*Power 3.1 [28].

### Study procedure

The participants sat with a data collector in a room where they could observe and evaluate the patient monitoring scenarios undisturbed. Before each session, participants completed a demographic survey, watched a 6-min educational video about Visual Patient Technology (Additional file 2: Video S1), and familiarized themselves with the layout of the conventional patient monitoring interface used in this study.


**Additional file 2 Video S1.** The instructional video that each participant in this study watched before data collection to learn how to monitor with Visual Patient technology.


**Fig. 1:**
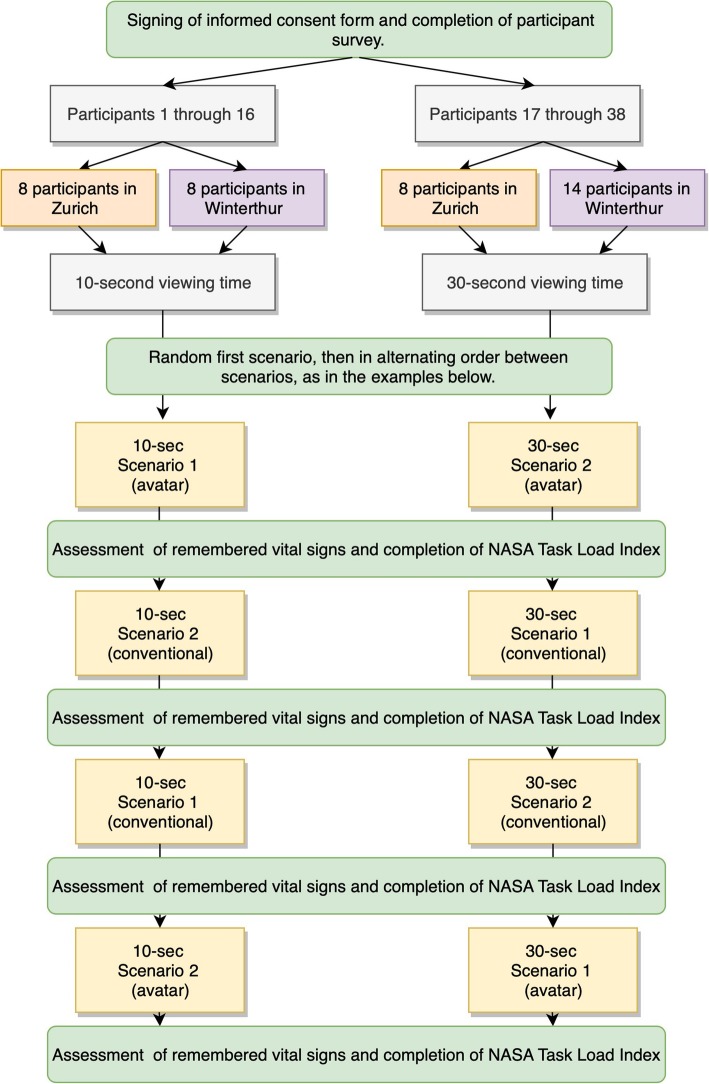
A flowchart showing the study procedure and examples of the interchanging order with which the scenarios were shown. NASA = National Aeronautics and Space Administration

For the study, each participant evaluated a total of four scenarios of either only 10-s or only 30-s duration. Figure 1 shows a flowchart of the study procedure. We showed participants the 10-s scenarios to simulate at-a-glance monitoring. At-a-glance, or observing the monitor in short glances, corresponds to real-life monitoring behavior of care providers as reported by several studies [7, 8]. We used 30-s scenarios to evaluate how the two monitoring technologies compared when users would be given significantly more time to observe and memorize a scenario. The four scenarios consisted of two identical scenarios, which were shown to each participant once in avatar-based and once in conventional form, hence the total of four scenarios shown. Participants were not aware of the two identical scenarios. The first scenario was picked at random and the following scenarios were presented in interchanging order between scenarios so that the same scenario never appeared twice in a row. The study design allowed us to compare the performance of each participant with the two different monitoring technologies, which allowed for intra-individual comparisons. After each scenario, participants had to name aloud the status of the vital signs they remembered of the critical patients. If a participant could not spontaneously name the status of all vital signs, the data collector queried the vital signs not yet actively mentioned by the participant. For example: “You have not yet named the status of respiratory rate for critical patient number 2, do you remember it? The participants had to choose between the response options “too high”, “normal”, “too low“ or “not perceived”. Also, after each scenario, participants completed the National Aeronautics and Space Administration (NASA) Task Load Index (TLX) questionnaire, a tool to measure perceived workload. It was originally developed for use in the aerospace industry, but has since been extensively used and validated also in human factors and healthcare research [29–37]. We found validation for the use of NASA TLX to measure subjective workload during patient monitoring tasks in previous studies. In one study, a standardized distraction by a calculation task increased the workload measured by NASA TLX in both avatar-based and conventional patient monitoring [25]. Also, in a previous study with a comparable setting, we found shorter observation times to be associated with higher NASA TLX scores [23]. We collected data for this study using an iPad-based data collection tool [38].

**Fig. 2:**
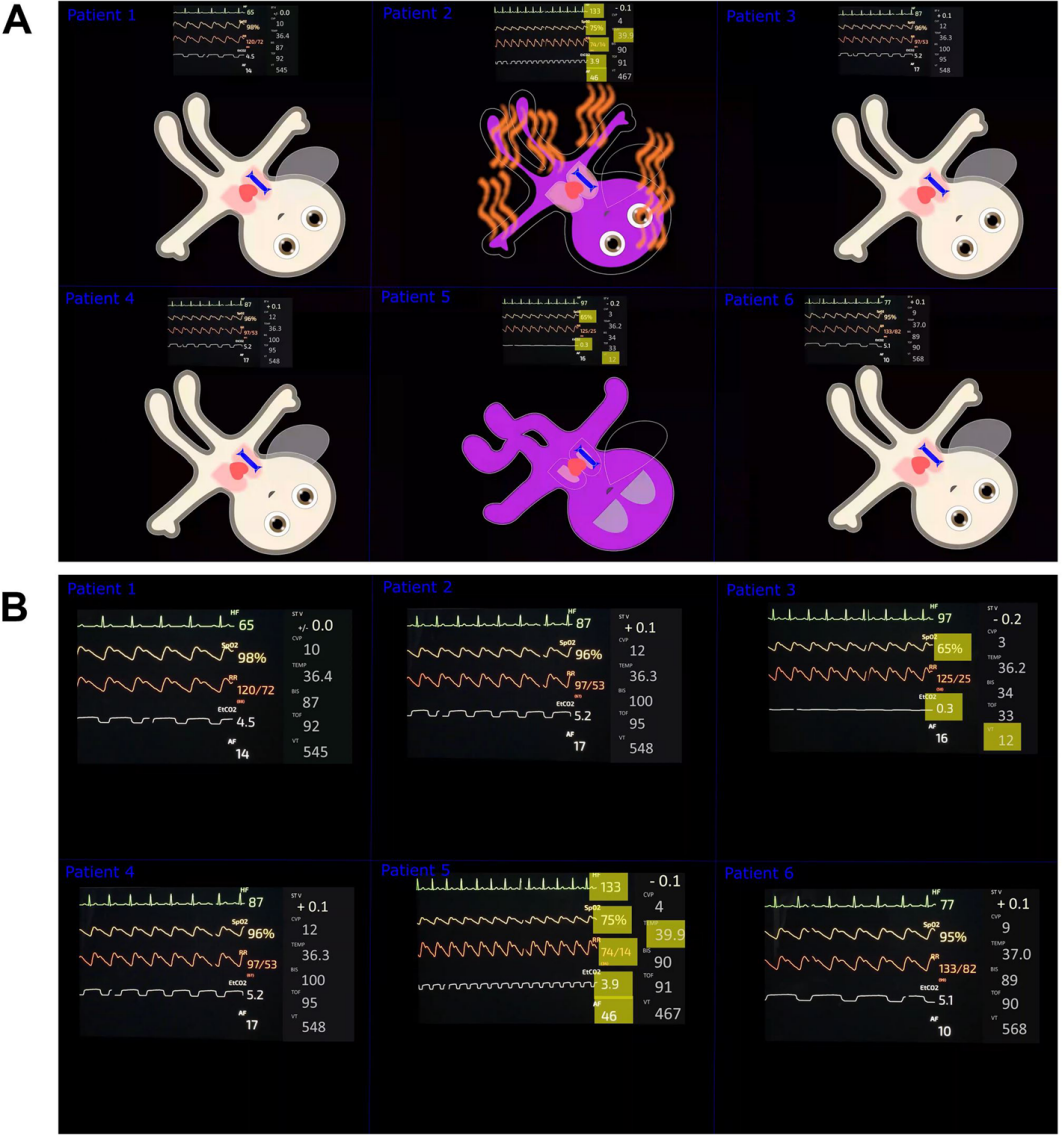
Scenario 1 showing a critical patient in septic shock and another patient with an endotracheal tube obstruction. **a** Avatar-based presentation. **b** Conventional, number and wave-form presentation

### Scenarios

To attain equal conditions for all participants, we recorded and played back the scenarios as videos. Figure 2 and Additional file 3: Video S2 shows the original scenarios used in this study with multiple patients represented at the same time. The conventional monitoring scenarios showed only a conventional number- and waveform-based monitor modelled after a GE Datex Ohmeda monitor (General Electric Company, Boston, MA, USA) using the simulator app SimMon (Castle 2 Andersen ApS, Hillerød, Denmark). The avatar-based scenarios showed the animated avatar in addition to the conventional display. To attain optimum realism, we highlighted the abnormal vital signs in all scenarios, as it is commonly used in modern day conventional patient monitors.

Per scenario, we showed six patients, two of which were critical with multiple vital sign abnormalities, and four of which did not show any vital sign abnormalities. For each patient, the following 11 vital signs were displayed: pulse rate, arterial blood pressure, oxygen saturation, central venous pressure, electrocardiogram ST-segment, respiratory rate, tidal volume, expiratory CO2 concentration, body temperature, brain activity, and neuromuscular conduction.

The critical patients in scenario 1 were a patient in septic shock and a patient with endotracheal tube obstruction. We simulated septic shock as pulse rate, respiratory rate and temperature too high, blood pressure, oxygen saturation and end-expiratory carbon dioxide concentration too low, other vital signs normal. We portrayed endotracheal tube obstruction as oxygen saturation, end-expiratory carbon dioxide concentration and tidal volume too low, other vitals normal.

The critical patients in scenario 2 were a patient in cardiopulmonary arrest and a patient with malignant hyperthermia. We simulated cardiopulmonary arrest as pulse rate, blood pressure, oxygen saturation, end-expiratory carbon dioxide concentration too low, ST-segment too high, other vital signs normal. We presented malignant hyperthermia as pulse rate, end-expiratory carbon dioxide concentration and body temperature too high, blood pressure too low, other vitals normal.

### Outcome measures

The primary objective of this study was to compare the perceptual performance of anesthesia providers observing central station patient monitor scenarios with both technologies. Therefore, we compared the number of vital signs participants were able to correctly recall with both monitoring technologies. A secondary objective was to compare the perceived workload during the task with both technologies. We evaluated perceived workload because high workload is a psychological stress factor. When workload demands exceed maximum human capacity, it interferes with our ability to remain aware of the situation at hand and will ultimately negatively affect decision making and task performance [3–7, 18, 35, 39]. In high workload situations, humans are susceptible to attentional tunneling and premature closure, the tendency to rush a decision without considering all aspects first. The goal of successful situation awareness design has been described as transferring the relevant information to the user with the least effort [4]. Another secondary endpoint of this study was the evaluation of the frequencies with which individual vital signs were correctly recalled by the participants.

### Statistical analysis

We express distribution of variables as medians with interquartile range (IQR) regardless of normality. To assess normality, we used the Shapiro-Wilks test and visual inspection of quantile-quantile plots of dependent variables. To compare perceptive performance and perceived workload between avatar-based and number and waveform-based monitoring scenarios, we used paired t-test as all data passed the normality test. We provide effect sizes as Cohen’s d. To assess differences between the study sites for statistical significance, we used Mann-Whitney or Fisher’s exact test as appropriate. We used GraphPad PRISM 8.1.1 (GraphPad Software Inc., CA, U.S.A.) for statistical analysis and creation of figures. A *p*-value of less than 0.05 was considered to indicate statistical significance.

## Results

Table 1 shows the study and participant characteristics. The samples from the two centers showed no statistically significant differences regarding occupational groups, gender, age or anesthesia experience.

**Table 1:** Study and participant characteristics

	KSW	USZ	*p*-value
Study duration in days (period)	12 (September 24th 2018 – October 5th 2018)		
Total number of participants	22	16	
Number of senior anesthetists (%)	6 (27%)	4 (24%)	*p* > 0.99
Number of resident anesthetists (%)	7 (32%)	6 (38%)	*p* = 0.74
Number of subspecialized nurse anesthetists (%)	9 (41%)	6 (38%)	*p* > 0.99
Number of female/male participants (%)	12 (55%)/10 (45%)	9 (56%)/7 (44%)	*p* > 0.99
Age of participants in years (%)	25–34: 7 (32)	25–34: 9 (56)	*p* = 0.084
	35–44: 9 (41)	35–44:6 (38)	
	45–54: 5 (23)	45–54: 0 (0)	
	54–65: 1 (4)	54–65: 1 (6)	
Anesthesia experience of participants in years (%)	1–5: 5 (23)	1–5: 8 (50)	*p* = 0.21
	5–10: 4 (18)	5–10: 4 (25)	
	> 10: 13 (59)	> 10: 4 (25)	

### Outcome measures

**Fig. 3:**
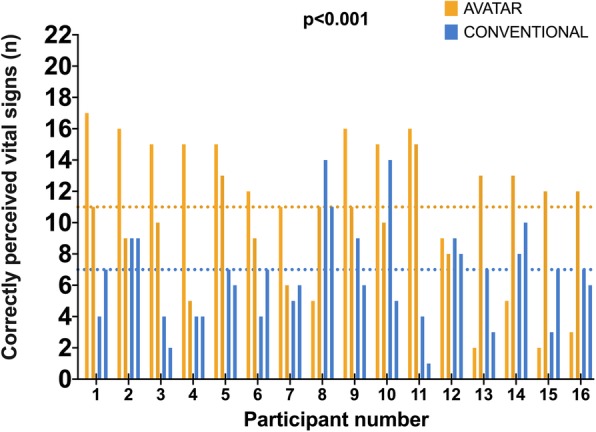
Perceptive performance in the 10-s scenarios. The dotted lines represent the median number of correctly perceived vital signs, which was 11 with avatar-based monitoring and 7 with conventional monitoring (paired Student’s t-test, *p* < 0.001, 95% confidence interval of the mean difference = 2 to 6 vital signs, effect size d = 0.67)

**Fig. 4:**
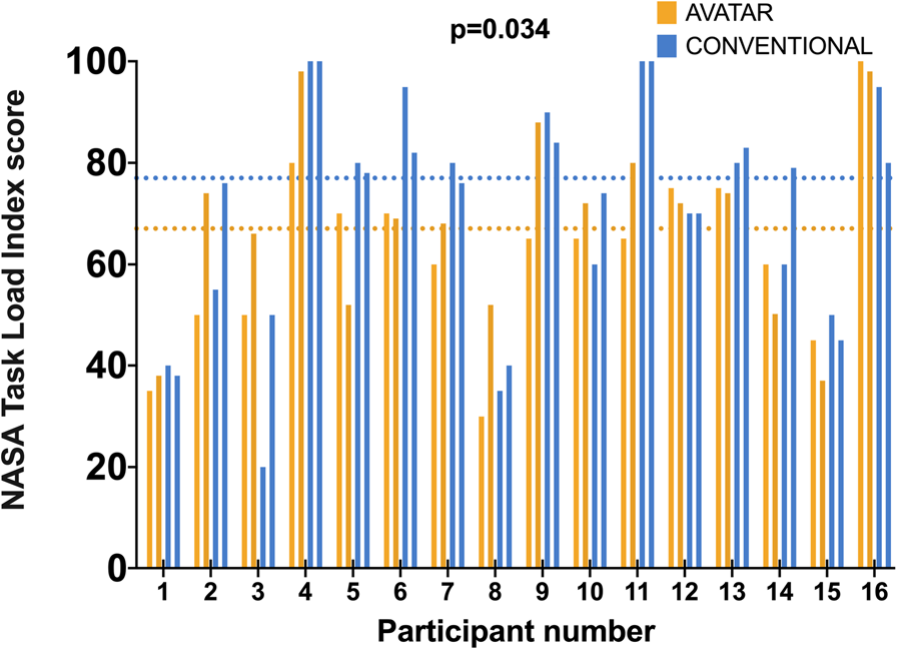
Perceived workload in the 10-s scenarios. The dotted lines represent the median NASA-TLX score, which was 67 with avatar-based monitoring and 77 with conventional monitoring (paired Student’s t-test, *p* = 0.034, 95% confidence interval of the mean difference = 0.5 to 11 points, effect size d = 0.43)

Figure 3 compares the anesthesia providers’ perceptual performance between avatar-based and conventional monitoring in the 10-s scenarios. Using avatar-based monitoring, anesthesia providers perceived a median of 11 (IQR 8 to 15) vital signs, compared to only 7 (IQR 4 to 9), using conventional monitoring, *p* < 0.001, mean of differences 4 vital signs, 95% confidence interval (CI) 2 to 6, effect size d = 0.67. Figure 4 compares the perceived workload in the 10-s scenarios. Using avatar-based monitoring, anesthesia providers had a lower median NASA TLX score of 67 (IQR 51 to 75), compared to 77 (IQR 51 to 84), using conventional monitoring, *p* = 0.034, mean of differences 6 points, 95% confidence interval 0.5 to 11, effect size d = 0.43. In the 30-s scenarios, the differences between the avatar and conventional monitoring were not statistically significant. Anesthesia providers perceived a median of 16 (IQR 13 to 18) vital signs with the avatar, vs. 15 (IQR 13 to 17), using conventional patient monitoring, *p* = 0.055, effect size d = 0.33. Likewise, NASA TLX scores did not differ in the more extended 30-s scenarios with a median score of 60 for both technologies, *p* = 0.59, effect size d = 0.13. Additional file 1: Figures S1 and S2 show these results. Additional file 1: Table S1 outlines the evaluation of the frequencies with which the individual vital signs were correctly recalled. In the 10-s scenarios, only two of 11 total vital signs, blood pressure and expiratory CO2 concentration, were perceived more frequently with conventional monitoring than with avatar-based monitoring. The other nine vital signs were correctly recalled more frequently or equally frequently with Visual Patient Technology.

## Discussion

Situation awareness errors may endanger patient safety, and therefore, maintaining an adequate situation awareness is a most important non-technical skill of anesthesia providers [2–7, 39]. This multicenter computer-based laboratory study focused on studying perception errors, which are the principal cause of situation awareness failures [3–6]. Anesthesia providers monitored multiple central monitor scenarios using an avatar-based virtual patient model and a conventional number- and waveform-based monitor. After an observation period, the computer screen darkened and participants had to recall patient status of the two critical patients shown in each scenario, and rate their perceived workload by completing the NASA TLX questionnaire.

Participants’ perceptive performance improved statistically and clinically significantly in the 10-s avatar-based scenarios by a mean of differences of 4 vital signs (95% CI 2 to 6). Perceived workload also decreased significantly by a mean of differences of 6 points in NASA TLX score (95% CI 0.5 to 11), compared to the 10-s conventional scenario. The observed effect sizes in these comparisons were medium to large [40]. This study was not powered to test for the statistical significance of the differences in the correct recall of individual vital signs. However, it still is remarkable that after only a short video introduction and at their first contact with the technology, the participants were able to recall 9 out of 11 vital signs more frequently than with regular monitoring, which they had been using daily for years.

The fact that the perceptive performance in the 30-s scenarios was not significantly different between the technologies suggests that avatar-based monitoring might be especially helpful when looking at monitors in short glances, which reflects the reality in clinical work [7, 8, 21]. The decreased size of the differences between avatar-based and conventional monitoring with increasing observation time, indicated by the smaller observed effect sizes in the 30-s scenarios, was similar to our previous studies, which investigated the effects of avatar-based monitoring for monitoring one patient at a time [23, 25, 26].

The reduced workload-levels participants perceived with avatar-based monitoring is of particular relevance, since central monitors are commonly located in areas with a high interaction between the observer and their environment, e.g. co-workers, noise, visitors, and phone calls [5]. Increased workload is associated with stress, perception errors and consequently impaired decision-making, all jeopardizing patient safety [1–6, 31, 35]. In this context, researchers identified inattentional blindness as a common form of perception error, which affects change detection and detection of unexpected events [9–14, 41].

### Limitations

This study was conducted as a computer-based laboratory study and as such has some specific limitations. Most importantly, this study did not test any patient outcome measures and only tested the technology in an undisturbed and simulated environment, which excluded potential effects of distractions that could be present in a real environment. It is, therefore, unclear how the results would translate into a real operating room or intensive care unit environment. Also, workload was measured with the NASA Task-Load-Index. Although this is a commonly used tool and, in our previous studies, we found indications of its validity in patient monitoring tasks, there are other, more direct, measures of stress, which we did not assess in this study, e.g., participants’ heart rate variability [42], pupil dilation [43], and blink frequency [44].

### Strengths

This multicenter study found a clinically and statistically significant performance improvement in the 10-s scenarios with Visual Patient Technology. The variety of anesthesia providers included into this study, with different professional experience and positions, as well as age and sex, increases external validity. The intra-participant design decreases potential effects of random noise.

## Conclusions

This study provides empirical evidence that avatar-based monitoring (Visual Patient Technology) in a central monitor station, when monitoring multiple patients at the same time, improves perception of vital sign information and reduces perceived workload, especially, when the monitor observation is of short duration. The next studies with the avatar-based technology will be conducted in a real or high fidelity simulated environment to factor in the effects of distraction, and assess direct measures of stress.

## Supplementary information

**Additional file 1: Table S1.** The numbers and percentages with which the participants correctly recalled the individual vital signs in the 10-s and 30-s scenarios. Each participant evaluated each vital sign a total of eight times (four times with either monitoring technology). In the 10-s scenarios, two of the 11 vital signs were more frequently recognized using conventional monitoring. In the 30-s scenarios, four of the 11 vital signs were more frequently recognized using conventional monitoring. **Figure S1.** Perceptive performance in the 30-s scenarios. The dotted lines represent the median number of correctly recalled vital signs, which was 16 with avatar-based monitoring and 15 with conventional monitoring (paired Student’s t-test, *p* = 0.055, effect size d = 0.33). **Figure S2.** Perceived workload in the 30-s scenarios. The dotted lines represent the median NASA-TLX score, which was 60 with avatar-based monitoring and also 60 with conventional monitoring (paired Student’s t-test, *p* = 0.59, effect size d = 0.13).

**Additional file 3. Video S2.** The multiple patient monitoring scenarios that the participants of this study evaluated.

## Data Availability

The datasets generated and analyzed during this study are available from the corresponding author on reasonable request.

## Abbreviations

IQR: Interquartile Range
KSW: Cantonal Hospital Winterthur
NASA: National Aeronautics and Space Administration
TLX: Task-Load-Index
USZ: University Hospital Zurich
