# Supplementary material for: Avatar-based versus conventional vital sign display in a central monitor for monitoring multiple patients: a multicenter computer-based laboratory study
Source: BMC Med Inform Decis Mak. 2020 Feb 10;20:26. doi: 10.1186/s12911-020-1032-4 (PMC7011453; doi:10.1186/s12911-020-1032-4)

**Avatar-based versus conventional vital sign display in a central monitor for monitoring multiple patients: A multicenter computer-based laboratory study.**

**Table S1:** The numbers and percentages with which the participants correctly recalled the individual vital signs in the 10-second and 30-second scenarios. Each participant evaluated each vital sign a total of eight times (four times with either monitoring technology). In the 10-second scenarios, two of the 11 vital signs were more frequently recognized using conventional monitoring. In the 30-second scenarios, four of the 11 vital signs were more frequently recognized using conventional monitoring.

|  | 10-second scenarios  N=16 | | 30-second scenarios  N=22 | |
| --- | --- | --- | --- | --- |
|  | conventional | avatar | conventional | avatar |
| Pulse rate (%) | 29 (45%) | 44 (69%) | 75 (85%) | 75 (85%) |
| Blood pressure (%) | 41 (64%) | 27 (42%) | 78 (89%) | 53 (60%) |
| Oxygen saturation (%) | 37 (58%) | 46 (72%) | 67 (76%) | 85 (97%) |
| Central venous pressure (%) | 1 (2%) | 24 (38%) | 45 (51%) | 55 (63%) |
| ECG ST-segment (%) | 13 (20%) | 21 (33%) | 58 (66%) | 58 (66%) |
| Respiratory rate (%) | 8 (13%) | 17 (27%) | 54 (61%) | 44 (50%) |
| Tidal volume | 15 (23%) | 15 (23%) | 68 (77%) | 57 (65%) |
| Expiratory CO2 concentration (%) | 35 (55%) | 25 (39%) | 66 (75%) | 51 (58%) |
| Brain activity (%) | 12 (19%) | 38 (59%) | 51 (58%) | 70 (80%) |
| Temperature (%) | 20 (31%) | 45 (70%) | 74 (84%) | 84 (95%) |
| Neuromuscular relaxation (%) | 6 (9%) | 33 (52%) | 43 (49%) | 54 (61%) |

**Figure S1:** Perceptive performance in the 30-second scenarios. The dotted lines represent the median number of correctly recalled vital signs, which was 16 with avatar-based monitoring and 15 with conventional monitoring (paired Student's t-test, p = 0.055, effect size d=0.33).


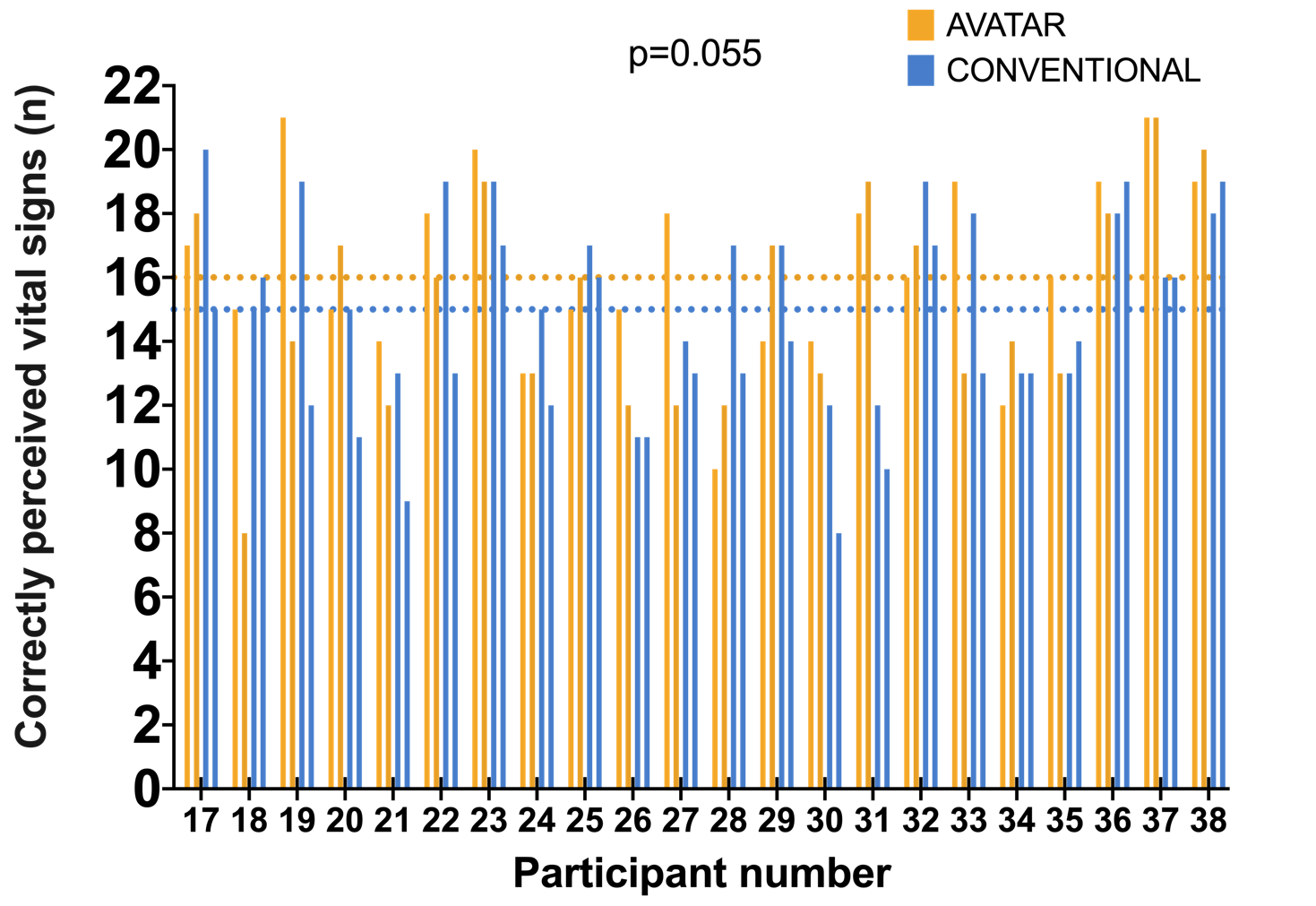


**Figure S2:** Perceived workload in the 30-second scenarios. The dotted lines represent the median NASA-TLX score, which was 60 with avatar-based monitoring and also 60 with conventional monitoring (paired Student's t-test, p = 0.59, effect size d=0.13).


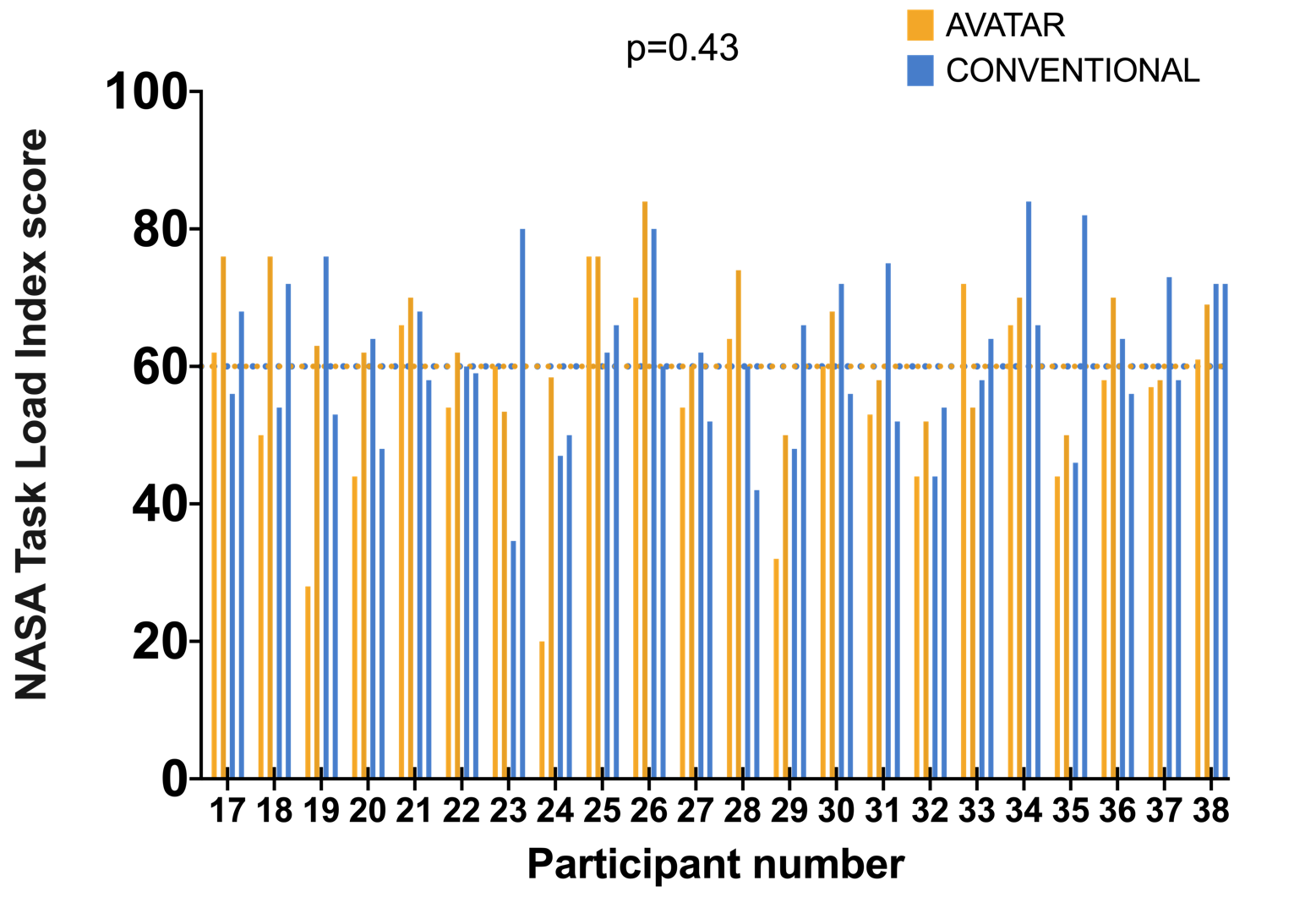

Supplement: Supplementary file 2 — Additional file 1: Table S1. The numbers and percentages with which the participants correctly recalled the individual vital signs in the 10-s and 30-s scenarios. Each participant evaluated each vital sign a total of eight times (four times with either monitoring technology). In the 10-s scenarios, two of the 11 vital signs were more frequently recognized using conventional monitoring. In the 30-s scenarios, four of the 11 vital signs were more frequently recognized using conventional monitoring. Figure S1. Perceptive performance in the 30-s scenarios. The dotted lines represent the median number of correctly recalled vital signs, which was 16 with avatar-based monitoring and 15 with conventional monitoring (paired Student’s t-test, p = 0.055, effect size d = 0.33). Figure S2. Perceived workload in the 30-s scenarios. The dotted lines represent the median NASA-TLX score, which was 60 with avatar-based monitoring and also 60 with conventional monitoring (paired Student’s t-test, p = 0.59, effect size d = 0.13). [file 12911_2020_1032_MOESM1_ESM.docx]
